# Size Matters: The Number of Prostitutes and the Global HIV/AIDS Pandemic

**DOI:** 10.1371/journal.pone.0000543

**Published:** 2007-06-20

**Authors:** John R. Talbott

**Affiliations:** Africans Against Aids, Inc., New York, New York, United States of America; Instituto de Medicina Tropical Alexander Von Humboldt, Peru

## Abstract

**Background:**

HIV/AIDS prevalence rates across countries of the world vary more than 500-fold from .06% in Hungary to 33.4% in Swaziland. One of the most cited research papers in the field, utilizing cross country regression analysis to analyze other correlates with this HIV prevalence data, is flawed in that it weights each country's results by the country's population.

**Methodology/Principal Findings:**

Based on cross-country linear and multiple regressions using newly gathered data from UNAIDS, the number of female commercial sex workers as a percentage of the female adult population is robustly positively correlated with countrywide HIV/AIDS prevalence levels. Confirming earlier studies, female illiteracy levels, gender illiteracy differences and income inequality within countries are also significantly positively correlated with HIV/AIDS levels. Muslims as a percentage of the population, itself highly correlated with country circumcision rates and previously found to be negatively correlated with HIV/AIDS prevalence, is insignificant when the percentage of commercial sex workers in a population is included in the analysis.

**Conclusions/Significance:**

This paper provides strong evidence that when conducted properly, cross country regression data does not support the theory that male circumcision is the key to slowing the AIDS epidemic. Rather, it is the number of infected prostitutes in a country that is highly significant and robust in explaining HIV prevalence levels across countries. An explanation is offered for why Africa has been hit the hardest by the AIDS pandemic and why there appears to be very little correlation between HIV/AIDS infection rates and country wealth.

## Introduction

One of the biggest puzzles facing HIV/AIDS researchers over the years is why some countries of the world have been so hard hit by the virus while others appear relatively unscathed. Some central and southern African countries are experiencing HIV/AIDS prevalence rates for their general populations in the 25% to 35% range while more than 40 other countries of the world report prevalence rates of .2% or less[1].

Most every variable suggested to explain Africa's high prevalence rates fails the simple test of asking why other countries outside Africa have not had a similar experience. If circumcision is the key, why hasn't HIV/AIDS spread faster in those other non-African countries of the world that are predominately uncircumcised? If relatively wealthier developing countries in Africa, such as South Africa and Botswana, seem to experience greater rates of infection, why don't the wealthier developing countries of Latin America like Argentina and Brazil experience similar high prevalence rates?

It has been well documented that high risk communities of a country's population are most liable to have high HIV/AIDS prevalence rates. Specifically, intravenous drug users, prostitutes and homosexuals have generally had higher prevalence rates for the illness. This paper seeks to answer how a general heterosexual population might become broadly infected. Country prevalence rates of 25% to 30% of the entire adult population can not be explained solely by the infection rates of these relatively small high risk groups. The paper does not address MSM (men having sex with men) directly as it represents its own community. Analyzing bisexual contact and possible viral transference between the MSM community and the heterosexual community is beyond the scope of this paper but may warrant further study.

One variable that appears to have a high degree of correlation with the infection rates for the general population is the reported HIV/AIDS prevalence rates for a country's commercial sex workers (CSW's). Detailed surveys of the infection rates for prostitutes have been performed in many of the countries of the world for the last twenty plus years. In general, even in the most infected countries, prevalence rates for CSW's appear to be significantly higher than the general populace and seem to be a leading indicator of future levels of prevalence in the general populace. However, the fit is not perfect and there are obvious feedback issues to consider, as you would expect CSW's to become infected eventually if operating in a high prevalence environment.[2], [3]

This paper recognizes the unusual power of CSW's to spread a sexually transmitted illness like HIV/AIDS.[4], [5], [6] CSW's have hundreds of new sexual partners each year while people in traditional monogamous relationships might not have any[7]. In addition, CSW's typically have much higher rates of sexually transmitted diseases (STD's) making HIV/AIDS transmission even more efficient. Also, many CSW's frequently inject themselves, and sometimes their clients, with intravenous drugs using dirty needles, a common mechanism of HIV/AIDS transmission.

It is not only the degree to which CSW's are infected in a country that is important or how easily they might transmit the virus, but also the sheer number of CSW's working in a country that must be weighed. CSW's represent a dramatically different percentage of the total female adult population across countries of the world (across our 77 country sample the range is from .05% to 10.0% of female adults, a 200-fold variance)[8], and yet this portion of the explanatory power of commercial sex work and HIV/AIDS has been missing to date. The primary reason is quite obvious. Countries of the world are very hesitant to speak about their HIV/AIDS exposure as it directly affects economic investment, tourism and perceived country status. They seem even more reluctant to measure and disclose the number of CSW's in their countries.

Combining the two variables, the number of prostitutes and their infection rates leads to a new third variable, the number of infected prostitutes in a country (expressed as a percentage of the total adult population age 15 to 49 years) which becomes very important in its correlation with HIV prevalence levels across countries, understanding that such a measure has feedback and endogeneity concerns associated with it..

In a widely cited paper, Drain et al (2004)[9], the authors performed similar cross country regression analyses across countries of the world to try to identify correlates with HIV prevalence. They made a serious error when they weighted their data by each country's population. In effect, they make data from China 400 times more weighted in their analysis than that from small population Botswana, an error that leads to many false conclusions. There is no reason to think that China's reported data is any more accurate than Botswana's and in trying to uncover possible government and social action that could correlate with HIV it makes no sense to weight one country's experiences or attempts more highly than another. More populous countries do not necessarily conduct more or better testing of their CSW populations. Halperin and Bailey (1999)[10] make the same error in their paper and incorrectly conclude that male circumcision is the key correlate with HIV prevalence rates across countries of the world. We correct this error in this paper by not weighting results by country population.

## Methods

Cross-country linear and multiple regression analyses were performed on data from 77 countries of the world[11]. The analysis was not limited to only the developing world as valuable information on correlates with HIV/AIDS prevalence is also available from the developed countries of the world.

The 77 countries that had data available and are included in our analyses are listed in Table 1 with each country's 2005 HIV/AIDS prevalence rate for its general population. The names assigned to the variables and their descriptions are presented in Table 2 followed by a list of the primary data sources for each variable.

**Table 1:** 77 Countries Included in Primary Analysis

**Caribbean and Central America**	**2005 HIV/AIDS Prevalence**
Dominican Republic	1.11
Guatemala	0.90
Haiti	3.81
Honduras	1.54
Panama	0.89
**South America**	
Argentina	0.61
Bolivia	0.13
Brazil	0.54
Colombia	0.61
Guyana	2.45
Peru	0.57
Venezuela	0.72
**Central Europe**	
Austria	0.29
Bosnia and Herzegovina	0.10
Czech Republic	0.10
Poland	0.12
Slovakia	0.10
**Eastern Europe**	
Armenia	0.15
Belarus	0.34
Latvia	0.79
Lithuania	0.17
Russian Federation	1.09
**Western Europe**	
Germany	0.12
Italy	0.50
Netherlands	0.22
Spain	0.62
United Kingdom	0.24
**Asia (Excluding China and India)**	
Cambodia	1.64
Indonesia	0.80
Japan	0.10
Korea, South	0.10
Laos	0.12
Malaysia	0.47
Nepal	0.53
Philippines	0.10
Sri Lanka	0.10
Tajikistan	0.14
Thailand	1.40
Turkmenistan	0.10
Viet Nam	0.51
**China and India**	
China	0.10
India	0.92
**Middle East and North Africa**	
Iran	0.15
Morocco	0.10
**Africa (Excluding North Africa)**	
Angola	3.68
Benin	1.79
Botswana	24.10
Burkina Faso	2.01
Cameroon	5.43
Central African Republic	10.73
Côte d'Ivoire	7.06
Democratic Republic-Congo	3.23
Djibouti	3.11
Eritrea	2.36
Ethiopia	2.30
Gambia	2.44
Ghana	2.27
Guinea	1.52
Kenya	6.09
Madagascar	0.51
Malawi	14.09
Mali	1.73
Mauritius	0.55
Mozambique	16.11
Namibia	19.56
Niger	1.10
Nigeria	3.86
Rwanda	3.07
Senegal	0.91
Sierra Leone	1.56
South Africa	18.79
Togo	3.24
Uganda	6.66
Zambia	16.96
Zimbabwe	20.12
**North America**	
Mexico	0.28
United States of America	0.30

**Table 2:** Names of Variables and Their Descriptions and Primary Sources

Name	Description of Variable	Primary Sources
**CSW** **[32], [33]**	Adult Commercial Sex Workers as a Percent of Female Adult Population (Ages 15 to 49)	See “Estimates of the Number of Female Sex Workers in Different Regions of the World” for extensive description of data collection methodology.
		J Vandepitte, R Lyerla, G Dallabetta, F Crabbé, M Alary and A Buvé
		Sexually Transmitted Infections 2006;82;18-25
**NUM**	Number of “HIV Infected” Commercial Sex Workers per 100,000 of Female Adult Population	(CSW*HIC)*10
**LOI**	Log of (NUM)	LOG(NUM)
**HIV** [**34**]	HIV Prevalence-General Population of Country	Annex 2-HIV and AIDS estimates and data, 2005 and 2003
		Source: 2006 Report on the global AIDS epidemic, UNAIDS/WHO, May 2006.
		Available at http://www.unaids.org/en/HIV_data/2006GlobalReport/default.asp
**HIC** [**35]**, [36****]	HIV Prevalence-Commercial Sex Workers	Weighted average of over 6,500 recent surveys of female prostitutes reported in the HIV/AIDS Surveillance Data Base at the Population Division, U.S. Bureau of the Census
		Available for download at http://www.census.gov/ipc/www/hivaidsd.html (Small surveys of less than 30 respondents were excluded as were surveys reported of low or unknown quality and weighting was by size of survey giving emphasis to more recent surveys that were national in scope.)
**MUS** [**37**]	Muslim Percentage of Total Country Population	U.S. Central Intelligence Agency's The World Factbook 2006
		Available at https://www.cia.gov/cia/publications/factbook/geos/bn.html#People
**GDP** [**38**]	2005 GDP per Capita-Purchasing Power Parity (PPP)	World Bank
		Available at http://www.answers.com/topic/list-of-countries-by-gdp-ppp-per-capita
**ILW** [**39**]	Illiteracy Rate (%), Women-Ages 15–24	United Nations Statistics Division
		Available at http://unstats.un.org/unsd/demographic/products/indwm/ww2005/tab4c.htm
**ILD**	Difference-Women's Minus Men's Illiteracy Rates (%)-Ages 15–24	United Nations Statistics Division
		Available at http://unstats.un.org/unsd/demographic/products/indwm/ww2005/tab4c.htm
**GIN** [**40**]	Gini Coefficient as a Measure of Income Inequality (Higher Gini signifies higher inequality)	United Nations 2005 Development Programme Report (page 270).
		Available in footnote at http://en.wikipedia.org/wiki/List_of_countries_by_income_equality
**POP**	Female Adult Population Age 15 to 49	UN Department of Economic and Social Affairs-Population Division
		Available by adding five-year sub age groups at http://esa.un.org/unpp/index.asp?panel = 2

The primary source of data on the number of commercial sex workers is a June 2006 published paper by Vandepitte et al[12] of UNAIDS. Because multiple sources were used in trying to estimate the number of commercial sex workers by country, the commensurate differences in the definition of commercial sex work leads to additional error in specifying the variable. CSW's were defined as women having sex in a formal exchange for money or goods, so male CSW's were ignored for the purposes of this paper. Percentages were taken as a percent of the adult female population in their sexually active years, defined by UNAIDS as women 15 to 49 years old. Direct (sex work as profession) as well as indirect (sex work on the side) commercial sex work was included in the definition, but infrequent and informal exchanges of sex for goods and services were not.[13]

A number of linear and multiple regressions were run to try to understand not only the correlates of these variables with HIV prevalence across countries of the world, but also their interdependence. Tests were conducted for omitted variable bias and attempts were made to see if causality direction could be implied from the data.

The first regression run is a simple one variable linear regression with the HIV/AIDS prevalence percentage for the general population of a country (HIV) as the dependent variable and the percentage of the population that are commercial sex workers (CSW) as the independent variable. Four new independent variables are added to a multiple regression to test for robustness of this variable against variables that were previously found to be important correlates with HIV prevalence.[14] (GIN, MUS, ILW, ILD). Because of the high collinearity between ILW and ILD[15], two separate regressions were run, one for each. To search for possible omitted variables we introduce a new variable, GDP, to the analysis and run new linear and multiple regressions to test its significance.

Next, we explore whether some of these variables aren't more likely to be correlated directly with, and possible causes of, CSW levels. We perform regression analysis on these variables, now with CSW as the dependent variable.

We introduce the rate of infection of the CSW population as a new variable named HIC, recognizing inherent feedback and endogeneity concerns with the HIV variable. We run two new regressions, one linear and one multiple, to test its significance in correlating with HIV. To control for possible interdependence between CSW and HIC we perform a principal components analysis of these two variables on HIV[16].

Finally we multiply CSW and HIC together to get a new variable, called LOI, which is the number of infected prostitutes or infected commercial sex workers in a country expressed as a percentage of the total adult population age 15 to 49 years. Two final regressions are run to demonstrate the high degree of correlation between this new variable and HIV, understanding that it suffers from the same endogeneity concerns as the HIC variable.

## Results

Table 3 presents a simple one variable linear regression with the HIV/AIDS prevalence percentage for the general population of a country (HIV) as the dependent variable and the percentage of the population that are commercial sex workers (CSW) as the independent variable. The variable CSW appears to be highly significant in this simple test as it attains a t-stat of 4.47 and the regression has an R-squared of 20%.

**Table 3:** Linear One-Variable Regression-HIV and CSW

Variable	Coefficient	Standard Error	t-stat
CSW	1.370477	0.306757	4.467632
Constant	0.585524	0.774109	0.756384
R-squared	0.210192		
Adjusted R-squared	0.199661		

HIV = +1.3704770143746 CSW +0.58552357299504+e

The robustness of this apparent correlation can be tested through multiple regressions presented in Table 4. We introduce four additional variables to the analysis that previously were found to be significant in predicting general population HIV/AIDS prevalence rates in both linear and multiple regression models.[17] The four new variables are the Gini coefficient (GIN), which is a measure of the inequality of incomes within a country, the percentage of a country's population that are of the Muslim faith (MUS), the percentage of a country's young adult (age 15 to 24 year old) women that are illiterate (ILW) and the difference between the young female and young male illiteracy rates (ages 15 to 24 years old) (ILD). Drain et al. tested over 80 variables and these four, of the variables that had enough data gathered to make meaningful comparisons, were the most significant in predicting HIV/AIDS levels in both linear and multiple regressions. Other variables that were found by Drain et al. to be significantly correlated with HIV/AIDS prevalence levels were either clearly measuring a result of high HIV prevalence such as life expectancy, or represented sexual behavior variables which seemed promising, but data was only available for a very few countries.[18]

**Table 4:** Two Multiple Regressions for Testing Robustness of CSW (One using ILW, the second using ILD)

Case 1-Variable	Coefficient	Standard Error	t-stat
CSW	0.227663	0.081709	2.786274
GIN	0.057345	0.013763	4.16654
MUS	−0.780485	0.508837	−1.533862
ILW	0.022013	0.007053	3.121285
Constant	−3.303085	0.587629	−5.621042
R-squared	0.486281		
Adjusted R-squared	0.457741		
**Case 2-Variable**	**Coefficient**	**Standard Error**	**t-stat**
CSW	0.231579	0.081812	2.830617
GIN	0.064668	0.013278	4.870187
MUS	−0.512487	0.469547	−1.09145
ILD	0.054215	0.01785	3.037196
Constant	−3.564639	0.576557	−6.182626
R-squared	0.483005		
Adjusted R-squared	0.454284		

Case 1 ln(HIV) = +0.22766264030035 CSW+0.057344715963125 GIN −0.78048548378733 MUS +0.022013331863842 ILW −3.3030854110271+e

Case 2 ln(HIV) = +0.23157932453245 CSW +0.064667778420059 GIN −0.51248737318915 MUS +0.054214879918507 ILD −3.5646387429584+e

The two multiple regressions presented in Table 4 demonstrate that the variable, CSW, the percentage of commercial sex workers in a country's population holds up quite well with reported t-stats of 2.79 and 2.83, respectively. GIN (t-stats of 4.17 and 4.87), ILW (3.12) and ILD (3.04) also perform well under this specification. All four significant variables have positive coefficients suggesting that increases in the percentage number of commercial sex workers in the country, increases in inequality, increases in female illiteracy and increases in the difference between young adult female and male illiteracy rates are all correlated with increases in the HIV/AIDS prevalence rate for a country's general population.

The only variable that performs poorly in both regressions in Table 4 is MUS, the percentage Muslims in the country's population. While some negative correlation appears to exist, the t-stats for MUS in the two regressions of −1.53 and −1.09 do not reach standards of significance. Previous research papers have reported a high degree of statistical significance between the Muslim faith and its negative correlation with HIV/AIDS prevalence rates in the general population of a country.[19] Some have suggested that this apparent decline in prevalence rates in more Muslim countries results from the fact that most Muslims are circumcised.[20] There have been numerous other research papers that have purported to show a correlation between circumcision and reduced HIV/AIDS viral transmission rates during heterosexual sex.[21], [22], [23], [24], [25], [26] It has been estimated that viral transmission rates may be reduced by as much as 60% through circumcision. Our results suggest that male circumcision may be overstated as a means of controlling a national AIDS epidemic

In our analysis, it appears that CSW, GIN, ILW and ILD all have valid correlations with HIV/AIDS and the adjusted R-squareds of the regressions suggest that approximately 45% of the variance in HIV has been explained by these variables. ILW might be measuring the difficulty illiterate females have in acquiring knowledge about the virus in order to take proper preventative action. ILD might be a crude measure of gender inequality in a society and therefore proxy for women's difficulties in attaining self-reliant incomes. Income inequality in a society may create an environment in which the richer prey on the poorer. Counter to conventional wisom, Gini coefficients and country inequality typically decrease as countries become wealthier (Roll and Talbott 2002)[27]. If, perhaps, Gini is acting as a proxy for country wealth, its positive correlation with HIV/AIDS levels suggests that HIV/AIDS levels would be negatively correlated with country wealth. We will come back to this later in this section.

To visualize the potential power of CSW's in spreading the virus, one need only assume that in a country with 4% of its adult females working as CSW's, if each CSW has sex with ten new male clients in a week, assuming no repeat customers in the week, this leads to contact with 40% of the adult male population in just one week. Not that all will get infected in the first week, but there are 51 more weeks to the year for repeat visits that, even given the low effective transmission rates of the virus, will most likely result in a huge expansion of the virus to the general public. Contrast this with purely monogamous relationships[28] in which there is zero new transmission or even casual sex relationships where people sleep with one to three others per year and you begin to understand the power of the CSW community to spread what is supposed to be a difficult disease to transmit.

The most likely reason for the CSW-HIV correlation if it is found that CSW is not a cause of HIV is that there exists a third, as yet unnamed, omitted variable that is highly correlated with both variables, CSW and HIV and in fact may cause both. If true, we would find high correlations between CSW and HIV and yet no true causality as both are being caused by the third factor. We have controlled somewhat for endogeneity by testing the CSW against variables that have been found in previous studies to have a high degree of correlation with HIV. Here, we will introduce additional variables that may in fact be the true causal variable.

The first variable to be tested is GDP per capita (GDP) as a measure of a country's wealth. One might expect poorer countries to have both higher HIV/AIDS prevalence rates and more economic motivation for poor women to enter prostitution and thus higher values for CSW. GDP per capita might be the omitted variable we referred to above that is driving both HIV and CSW and causing a possible illusion of causality between the HIV and CSW variables.

In Table 5, we present one linear and two multiple regressions that introduce GDP to the analysis. It was omitted purposely in Table 4 because of its strong negative correlation with GIN and the fact that previous researchers have been unable to uncover a strong relationship between GDP and HIV. Some have reported a positive relationship while others have reported a negative relationship. The regressions demonstrate some negative correlation between GDP and HIV, but the significance disappears when GDP is asked to compete against the previous variables tested. The sign of the coefficient of GDP is negative which suggests that indeed, greater country wealth is negatively correlated with higher HIV prevalence rates. But in the multivariable regressions GDP loses its significance and adds nothing to the adjusted R-squared.

**Table 5:** Tests for Addition of GDP per Capita as Significant Variable

Case 1-Variable	Coefficient	Standard Error	t-stat
GDP	−7.5E-05	1.8E-05	−4.155919
Constant	0.48553	0.221256	2.194426
R-squared	0.187183		
Adjusted R-squared	0.176345		
**Case 2-Variable**	**Coefficient**	**Standard Error**	**t-stat**
CSW	0.345284	0.088453	3.90359
GDP	−6E-05	1.7E-05	−3.542691
Constant	−0.250761	0.276985	−0.905322
R-squared	0.325977		
Adjusted R-squared	0.30776		
**Case 3-Variable**	**Parameter**	**Standard Error**	**t-stat**
MUS	−0.858176	0.512807	−1.673485
CSW	0.221763	0.081713	2.713912
GIN	0.052808	0.01432	3.687719
ILW	0.019151	0.007488	2.557652
GDP	−2E-05	1.8E-05	−1.118881
Constant	−2.855268	0.71002	−4.021388
R-squared	0.495336		
Adjusted R-squared	0.459796		

Case 1 ln(HIV) = −7.4812131154824E-05 GDP +0.48553005215236+e

Case 2 ln(HIV) = +0.34528384149589 CSW −5.9991903298253E-05 GDP −0.25076059417652+e

Case 3 ln(HIV) = −0.85817576092473 MUS +0.22176255497681 CSW +0.052807524890694 GIN +0.019150836576975 ILW −2.0275607109274E-05 GDP −2.8552678760593+e

GDP per capita may be poorly correlated with HIV prevalence rates across countries because of countervailing influences. People in poorer countries may be more desperate, less educated and less forward thinking making HIV/AIDS spread easier. But wealthier, rapidly growing developing countries may have their own set of issues making HIV/AIDS more prevalent. Wealthier developing countries typically have more intra- and inter-country commerce and better highway systems making HIV transmission easier. The number of people enjoying the anonymity of city life, conducive to an aggressive sexual lifestyle, usually increases as countries develop with many leaving the stable agrarian village life behind. Development, typically, means more job specialization and therefore more susceptibility for unskilled women to become unemployed. Finally, mining and other industrial employment common in development, in Africa at least, means more migrant workers living on their own making them more susceptible to multiple sexual encounters and prostitution.

CSW holds up quite well and reports a t-stat in the multivariable regression of 2.71 when GDP is introduced to the analysis. This suggests that while country wealth may have something to do with HIV levels in the country, it does not appear that GDP per capita is the missing omitted causal variable that is driving both CSW and HIV. CSW has significant correlation with HIV even after controlling for GDP.

To gain a deeper understanding of causality, it may be helpful to explore which variables are correlated with, and may have potential causal relationships with the level of CSW. This regression analysis is performed in Table 6. Unlike our previous attempts at explaining HIV levels, now MUS (-) and again ILW (+) are significant in explaining CSW, while GIN and GDP have little to no significance. The Muslim faith had no direct significant correlation with HIV levels, but it is significant in predicting the number of commercial sex workers in a country. The illiteracy rate in women is not only a predictor of HIV prevalence in a general population, but is also correlated with a higher number of commercial sex workers in the country. We now have a more complete understanding of how the Muslim faith and female illiteracy impacts HIV/AIDS prevalence rates, partly they determine the number of commercial sex workers in a country which then impacts the general HIV/AIDS prevalence rate, but it is true that even after controlling for CSW, female illiteracy has its own independent correlation with HIV.

**Table 6:** Correlates with Number of Commercial Sex Workers (CSW's)

Variable	Coefficient	Standard Error	t-stat
MUS	−0.897059	0.396015	−2.265217
GIN	0.011802	0.010844	1.088278
ILW	0.015621	0.005761	2.711373
GDP	0	1.4E-05	0.032379
Constant	−0.485873	0.551491	−0.881016
R-squared	0.169921		
Adjusted R-squared	0.123805		

ln(CSW) = −0.89705937218413 MUS +0.011801579240931 GIN +0.01562070546252 ILW +4.547389453781E-07 GDP −0.48587259240769+e

Of course, the shear number of CSW's in a country are only part of the story in understanding how big an impact the CSW community can have on HIV/AIDS infection rates in the general community. Perhaps, equally as important in determining the infection rate for an entire country is the infection rate of its CSW population. The percentage of CSW's infected with the HIV/AIDS virus is named here HIC[29]. We have purposely avoided bringing HIC into our formal analysis to date because of the inherent problems this variable has with endogeneity and feedback. A country with higher infection rates in its CSW population will most likely eventually suffer higher infection rates in the general population if there is a sufficiently large enough CSW community.[30] But, there can be no assurance from a statistical standpoint, that it is not the high level of infection rates in the general population that is indeed causing the high rates of infection in the CSW community. Therefore, we cannot prove that correlations between HIC and HIV are genuine and feedback free, but we would be remiss from a policy perspective if we did not at least discuss the potential impact of a highly infected CSW community.

**Figure 1:**
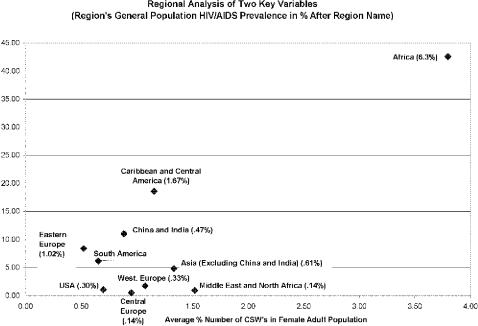
Regional Analysis of Two Key Variables.

If ever you have asked the question, how is Africa unique from the rest of the world, in a way that might explain the high HIV/AIDS prevalence rates in many of its countries, you need look no further than Figure 1. Regardless of the degree to which you think CSW and HIC accurately predict or cause HIV levels in the general population, a look at Figure 1 will at least make one realize that with regard to these two variables, CSW and HIC, Africa is indeed unique. On average, Africa has more than four times as many CSW's as the rest of the world (as a percent of the population) and the CSW community in Africa is more than four times as infected with HIV/AIDS as the rest of the world. Again, in a world in which Africa's experience with HIV/AIDS is so uniquely different from the rest of the world, such uniqueness measured by these two variables is an indication of their potential appeal as explainers of the crisis on the African continent. It is hard to imagine another variable in which Africa is so unique from the rest of the world, especially one that is as powerful in the physical dynamics of HIV/AIDS transmittance as the CSW community.

Table 7 and Table 8 demonstrate the powerful correlations between HIV and HIC, realizing that part of the reported correlation is due to uncontrollable feedback from the dependent variable to the independent variable. Ignoring obvious endogeneity concerns yields an explanatory adjusted R-squared for these variables of 75%.

**Table 7:** Linear One Variable Regression–HIV and HIC

Variable	Coefficient	Standard Error	t-stat
HIC	0.059493	0.004497	13.230405
Constant	−1.321649	0.136612	−9.674455
R-squared	0.700052		
Adjusted R-squared	0.696053		

ln(HIV) = +0.05949255801635 HIC −1.3216490064977+e

**Table 8:** Multiple Regression for Testing Robustness of HIC

Variable	Coefficient	Standard Error	t-stat
HIC	0.050991	0.00504	10.1166
GIN	0.039913	0.009418	4.237893
MUS	−0.193327	0.351101	−0.55063
ILW	0.00122	0.005302	0.230073
Constant	−2.883247	0.401433	−7.182392
R-squared	0.764471		
Adjusted R-squared	0.751386		

ln(HIV) = +0.050990873365278 HIC +0.039913198898174 GIN −0.19332672529913 MUS +0.0012197858724134 ILW −2.8832474794526+e

In order to control for multi-collinearity between the independent variables, a principal components analysis[31] of CSW and HIC as independent variables and HIV as the defendant variable is run and results are reported in Table 9. The fact that CSW maintains its significance in the linear term in competition with HIC buttresses the argument that the sheer number of CSW's is an important variable because we know that HIC benefits from unfair feedback from the HIV dependent variable. Obviously, the explanatory value of the HIC variable is being overstated due to feedback from the dependent variable, HIV, so the adjusted R-squared reported of 74% should not be viewed as a statistically rigorous test.

**Table 9:** Principal Components Analysis Original Coefficients Transformed Back From Principal Components Dependent Variable is HIV (L is Linear Term–Q is Quadratic Term)

NAME	COEFFICIENT	ERROR	T-RATIO 74 DF	P-VALUE	CORR.
(CSW)L	0.31303	0.1513	2.069	0.042	0.234
(CSW)Q	−0.12889	0.1418	−0.9088	0.366	−0.105
(HIC)L	1.2049	9.64E-02	12.5	0	0.824
(HIC)Q	−1.2304	9.56E-02	−12.87	0	−0.831
CONSTANT	1.1602	0.1732	6.697	0	0.614
R-SQUARE	0.7501				
R-SQUARE ADJUSTED	0.7433				

Mathematically, the two variables CSW and HIC can be combined easily by multiplying them. The percentage of CSW's in a population (CSW) multiplied by the HIV/AIDS prevalence rate for the CSW subpopulation (HIC) equals the number of “infected” CSW's expressed as a percent of the total general population (a new variable named here LOI and measured in Log terms). As expected, LOI is so highly correlated with HIV in Table 10 and Table 11 that it singly explains 71% of the variance in HIV and dominates all the other suggested independent variables. Only GIN, a measure of inequality in society, retains its significance.

**Table 10:** Linear One Variable Regression–HIV and LOI

Variable	Coefficient	Standard Error	t-stat
LOI	1.097885	0.08024	13.682539
Constant	−2.03837	0.172501	−11.8166
Multiple R	0.844969		
R-squared	0.713972		

ln(HIV) = +1.0978848712563 LOI −2.038369742493+e

**Table 11:** Multiple Regression for Testing Robustness of LOI

Variable	Coefficient	Standard Error	t-stat
LOI	1.020558	0.095434	10.693818
GIN	0.041581	0.009032	4.603916
MUS	0.303164	0.350474	0.865014
ILW	−0.007334	0.005479	−1.338496
Constant	−3.646523	0.385178	−9.467115
R-squared	0.780122		
Adjusted R-squared	0.767907		

ln(HIV) = +1.0205576925391 LOI +0.041581175379939 GIN +0.3031643950786 MUS −0.0073336345551375 ILW −3.6465234042304+e

## Discussion

We have described a fairly robust correlation between the number of commercial sex workers as a percentage of a country's population and the HIV/AIDS prevalence rate for the country's entire population. We have tried to disclose the dangers of presuming that such an analysis proves causality. We presented descriptive statistics demonstrating how unique Africa is from the rest of the world with regard to not only its high prevalence rates of HIV/AIDS, but also its number of CSW's and its high HIV/AIDS infection rates in the CSW community. We saw that many highly infected countries in Africa had both a high percentage of commercial sex workers in their population and an unusually high degree of infection among these commercial sex workers.

Evidence has been presented that suggests that once the number of commercial sex workers in a country is controlled for, the percentage of the population that is of the Muslim faith is statistically insignificant in predicting HIV/AIDS prevalence rates in the general population. At a macro level, this suggests that circumcision rates should also be less significant across countries in predicting HIV/AIDS levels once varying levels of commercial sex work are included in the analysis as there is a very high correlation between circumcision and the Muslim faith percentage. Finally, we have presented evidence that there is little correlation between the wealth of countries and HIV/AIDS across the countries of the world, certainly among those countries that we examined that have shown more reason to monitor the illness closely.

It is unfortunate that much of the analysis of this paper has focused solely on the female commercial sex worker. As stated previously, for every heterosexual contact by a female commercial sex worker, there is a male partner. These males are the plausible conduit that spreads the HIV/AIDS infection out of the narrow CSW community and into the general population. They are husbands and boyfriends and men and boys of all ages. Many insist that their brides are virgins, that their wives are monogamous and their daughters avoid pre-marital sex, yet they exchange money for sex and do nothing to prevent their sisters and daughters from prostituting themselves. In examining the role of the commercial sex industry in explaining country HIV/AIDS infection rates we may uncover the real reason for the pandemic, a breakdown of the responsibilities of a brother to his sister, a father to his daughter and a husband to his wife.
